# Down-regulation of Lsm1 is involved in human prostate cancer progression

**DOI:** 10.1038/sj.bjc.6600163

**Published:** 2002-03-18

**Authors:** S Takahashi, S Suzuki, S Inaguma, Y-M Cho, Y Ikeda, N Hayashi, T Inoue, Y Sugimura, N Nishiyama, T Fujita, T Ushijima, T Shirai

**Affiliations:** First Department of Pathology, Nagoya City University Medical School, 1 Kawasumi, Mizuho-cho, Mizuho-ku, Nagoya 467-8601, Japan; Department of Urology, Aichi Cancer Center, 1-1 Kanokoden, Chikusa-ku, Nagoya 464-0021, Japan; Department of Urology, Nagoya Memorial Hospital, 4-305 Hirabari, Tenpaku-ku, Nagoya 468-8520, Japan; Carcinogenesis Division, National Cancer Center Research Institute, 5-1-1 Tsukiji, Chuo-ku, Tokyo 104-0045, Japan

**Keywords:** Lsm1, prostate cancer, tumour progression

## Abstract

Elucidation of genetic alterations is an approach to understanding the underlying molecular mechanisms of progression of human prostate cancers. We have searched for genes differentially expressed in advanced prostate cancers using cDNA-representational difference analysis, and thereby isolated the Lsm1 as one of down-regulated gene. An Lsm1 expression vector was transfected into PC3 cells, normally featuring down-regulated Lsm1, and four transfectants were established. No differences in morphology or cell proliferation were evident in comparison with parent PC3 or PC3/mock-transfectants. In contrast, significant suppression of invasive potential or metastatic ability of Lsm1 transfectants was observed in the Matrigel chemoinvasion assay and in nude mice, respectively. With human prostate cancers, almost all of informative prostatectomised cases without neoadjuvant therapy showed allelic retention in the Lsm1 region, whereas refractory cancers frequently showed allelic loss in this region. No critical gene mutations were found in open reading frame of Lsm1 in prostate cancers examined by PCR–SSCP analysis, including localised and refractory cancers. These results suggest that Lsm1 is deeply involved in prostate cancer progression through its down-regulation, independent of any gene mutation.

*British Journal of Cancer* (2002) **86**, 940–946. DOI: 10.1038/sj/bjc/6600163 www.bjcancer.com

© 2002 Cancer Research UK

## 

Prostate cancer has become the most common malignancy in men in western countries (Pisani *et al*, 1993) and while the incidence and mortality from this cause in Japan are much lower, they appear to be rapidly increasing (Hsing *et al*, 2000). Androgen ablation therapy is generally accepted for prostate cancer. However, the outgrowth of androgen-independent cancer cells is a frequent outcome, eventually lead to the patient's death. Therefore, understanding of the molecular mechanisms of the acquisition of metastatic potential or the androgen-independent phenotype of tumour cells is urgently needed.

Several molecular cytogenetic studies have demonstrated that interstitial deletions of chromosomes 5q, 6q, 7q, 8p, 10p, 10q, 13q, 16q, 17p, 17q, and 18q are the most prevalent changes in human prostate cancers (Latil and Lidereau, 1998; Brothman *et al*, 1999; Verma *et al*, 1999; Ozen and Pathak, 2000), suggesting the presence of tumour suppressor genes in these regions. In particular, loss of heterozygosity (LOH) at chromosome 8p has been found in a majority of prostate cancers.

We examined the genes differentially expressed in advanced prostate cancers using cDNA-representational difference analysis (RDA), which is an efficient and reliable technique to isolate genes differentially expressed between samples to be compared (Hubank and Schatz, 1994), and thereby isolated the Lsm1 gene as one of them. The Lsm1 gene was mapped to 8p11.2, a region which is frequently deleted in prostate cancers, using BLAST search program and down-regulation shown for refractory cancer and androgen-independent DU145 and PC3 cells. The data prompted the present examination of whether Lsm1 is a target gene involved in the acquisition of androgen-independence or the metastatic phenotype in human prostate cancers.

## MATERIALS AND METHODS

### Samples

Normal prostate and organ-confined prostate cancers were obtained from patients who underwent total cystoprostatectomy or prostatectomy for bladder or prostate cancer. None of these cancer patients had previously undergone chemotherapy, radiotherapy or hormonal therapy. Refractory prostate cancers were obtained from autopsy cases who died of the disease. All autopsy cases had undergone hormonal therapy using anti-androgens, LH–RH-agonist or castration. Human prostate cancer cell lines, DU145, LNCaP and PC3, were purchased from the American Type Culture Collection. For cDNA–RDA, localised prostate cancer (moderately differentiated adenocarcinoma, Gleason's score: 3+4=7) and refractory cancer (poorly differentiated adenocarcinoma, Gleason's score: 5+5=10) were used.

### cDNA–RDA

cDNA was synthesised by using molony murine leukemia virus reverse transcriptase (Gibco – BRL) with oligo-d(T) primers. Subsequent RDA was essentially performed with minor modifications as previously described (Ushijima *et al*, 1997). Briefly, to prepare representations (amplicons), 5 μg aliquots of double-stranded cDNA were digested with *Sau*3AI (Toyobo Biochemicals, Osaka, Japan), phenol extracted and ethanol precipitated, followed by ligation with R-Bgl adaptor cassettes (R-Bgl-24: 5′-AGCACTCTCCAGCCTCTCACCGCA-3′; R-Bgl-12: 5′-GATCTGCGGTGA-3′). The ligation products were amplified using the R-Bgl-24 as the primer (25 cycles of 1 min each at 95°C and 3 min at 72°C). Both tester and driver representations were digested with *Sau*3AI to remove the R-Bgl adaptors. The tester representations were then purified by gel filtration chromatography (cDNA spun column, Amersham Pharmacia Biotech) and then ligated to the J-Bgl adaptor (J-Bgl-24: 5′-ACCGACGTCGACTATCCATGAACA-3′; J-Bgl-12: 5′-GATCTGTTCATG-3′). Two hundred nanograms of J-adaptor-ligated tester DNA was mixed with 40 μg of the driver DNA, hybridised for 21 h at 67°C, and amplified by PCR with J-Bgl-24 as primer for 10 cycles. After digestion of single-stranded DNA with Mung-bean nuclease (New England Biolabs, Inc., Beverly, MA, USA), additional PCR amplification for 20–30 cycles with J-Bgl-24 was performed. The second competitive hybridization was performed by switching to the N-Bgl adaptor (N-Bgl-24: 5′-AGGCAACTGTGCTATCCGAGGGAA-3′; N-Bgl-12: 5′-GATCTTCCCTCG-3′). One hundred nanograms of ligation solution were mixed with 20 μg of driver DNA. Denaturing, reannealing, and selective amplification of the self-annealed product were performed as for the first cycle. The final PCR products which removed the adaptor were ligated into pBluescript II that had been digested with *Bam*HI (Toyobo Biochemicals) and treated with calf intestinal alkaline phosphatase (Takara Biomedicals, Shiga, Japan). After transformation of XL1Blue-competent cells, insert-positive plasmid clones were selected by PCR amplification of the inserts using T3 and T7 primers and restriction-digestion of the PCR products with *Sau*3AI.

### Sequencing analysis

DNA sequencing was performed on an ABI PRISM 310 Genetic Analyzer (Perkin Elmer Applied Biosystems) using a BigDye Terminator Cycle Sequencing FS Ready Reaction Kit (Perkin Elmer Applied Biosystems). Sequencing data were analysed with BLAST search program (http://www.ncbi.nih.gov/blast/blast.cgi) (Altschul *et al*, 1990).

### Northern blot analysis

Total RNAs were isolated using RNAzol (Tel-test, Inc., TX, USA) and 10 microgram aliquots from prostate tissues were separated by electrophoresis in 1% agarose-formaldehyde gel and transferred to nylon membranes (Hybond-N, Amersham Pharmacia Biotech, UK). RDA clones inserted into pBluescript II and a human glyceraldehyde-3-phosphate dehydrogenase (GAPDH) cDNA (Clontech, UK), employed as internal standard, were used as the probes. Expression signals were quantitatively analysed using a densitometer (ImageMaster, Amersham Pharmacia Biotech).

### DNA transfection

A human Lsm1 cDNA fragment including full open reading frame was cloned into the pTarget mammalian expression vector (Promega, WI, USA). DNA transfection into PC3 cells was carried out with a Lipofectin protocol (Life Technologies, Inc. MD, USA). Stable transfectants were selected with 500 μg ml^−1^ G418 (Life Technologies, Inc.) and established by ring cloning.

### Cell proliferation assay

PC3 and its transfectants were maintained in F-12K medium (Life Technologies, Inc.) containing 10% foetal bovine serum and incubated at 37°C under a humidified 5% CO_2_ atmosphere. Cells were seeded into a 24-well plate at a density of 1×10^4^ cells per 2 ml medium, and incubated for 3, 5 and 7 days. Live cells from each culture were counted using Trypan blue staining. Each assay was carried out at least in triplicate.

### Chemoinvasion assay

A membrane invasion culture system was used to measure tumour cell invasion (Albini *et al*, 1987; Hendrix *et al*, 1987). Sterile cell culture inserts with 8 μm pore sized polyethylene terephthalate filters (Becton Dickinson, NJ, USA) were coated with 100 μg basement membrane Matrigel (Becton Dickinson), dried under laminar flow hood, and placed in 24-well plates filled with F-12K medium containing 0.1% bovine serum albumin (BSA) and 10 μg ml^−1^ fibronectin as a chemoattractant. Cells (1×10^5^), suspended in F-12K containing 0.1% BSA, were seeded in cell culture inserts and, after incubation at 37°C for 24 h, the filters were fixed in 80% ethanol and stained with Giemsa. Invaded cells were counted under microscope at high power fields. Each assay was carried out at least in quadruplicate.

### Animals

Male KSN (nu/nu) nude mice, 5-weeks-old, were purchased from Japan SLC, Inc. (Shizuoka, Japan). The animals were maintained and treated in accordance with institutional guidelines and in compliance with national and institutional law and politics. Cells (1×10^6^) were inoculated into the subcutaneous tissues in their flanks. Tumour volume was calculated from the formula: tumour volume=(width)^2^×length/2 (Osborne *et al*, 1985). Mice were killed at 20 weeks for histological assessment of invasion or metastases of tumour cells.

### mRNA* in situ* hybridisation

A fragment of Lsm1 complementary DNA, corresponding to nucleotides 434 to 582 of Lsm1 cDNA (Salgado-Garrido *et al*, 1999), was cloned into the vector pGEM-T (Promega, Madison, WI, USA). Antisense and sense Lsm1 riboprobes were prepared using pGEM-T/Lsm1 as the template and a digoxigenin RNA labelling kit (Roche Diagnostics). After deparaffinisation, sections were treated with 3 μg ml^−1^ proteinase K for 20 min at 37°C, and postfixed in 4% paraformaldehyde for 10 min. The sections were then placed in hybridisation buffer containing digoxigenin-labelled antisense riboprobes for 16∼18 h at 42°C. After hybridisation, the sections were washed in 2×SSC/50% formamide for 30 min at 42°C, then TNE buffer (10 mM Tris-HCl (pH 8.0), 500 mM NaCl, 1 mM EDTA) for 10 min at 37°C. Thereafter, the sections were treated with 10 μg ml^−1^ of RNase (Roche Diagnostics) for 30 min at 37°C. The slides were rinsed in buffer A (100 mM maleic acid, 150 mM NaCl, pH 7.5) for 10 min, and then incubated with buffer C (1% blocking reagent (Roche Diagnostics) dissolved in buffer A). Horse-radish peroxidase conjugated sheep anti-digoxigenin Fab fragments (1 : 100, Roche Diagnostics) were applied for 30 min. The slides were then washed in buffer A followed by incubation with biotinylated tyramide in amplification diluent (NEN Life Science Products, Inc., Boston, MA, USA) for 10 min, three times in buffer A for 5 min and alkaline phosphate conjugated streptoavidin (1 : 5000, Roche Diagnostics) for 30 min. After sequential washing in buffer A containing 0.2% Tween 20 and buffer B (100 mM Tris-HCl (pH 9.5), 100 mM NaCl, 50 mM MgCl_2_), hybridization signals were detected with 5-bromo-4-chloro-3-indolyl-phosphate (BCIP)/4-nitro blue tetrazolium chloride (NBT) solution (Roche Diagnostics). The specificity of the *in situ* hybridisation was proven by parallel hybridisation of the sections with sense riboprobes.

### LOH analysis

LOH was examined by PCR amplification of highly polymorphic microsatellite repeat markers on chromosome 8p11.2, including D8S1747, D8S416 and D8S1722, around Lsm1 gene. The order of these loci has been determined by available human genome sequencing data for 8p11.2 of the Japan Science Technology Corporation (JST) (http://www-alis.tokyo.jst.go.jp/HGS/top.pl) to be: D8S1722-Lsm1 gene-D8S416–D8S1747. PCR reactions were performed with ^32^P-end-labelled primers for 35–45 cycles at 94°C for 1 min, 56°C for 1 min and 72°C for 1 min in a 15 μl volume. The PCR products were separated on 6% polyacrylamide sequencing gels and used to expose X-ray films. Allelic loss was judged when a reduction in signal intensity of ⩽50% in one allele was evident in the tumour as compared to the paired normal tissue.

### Gene mutation analysis

The region covering Lsm1 coding sequences was amplified by PCR in the presence of [α-^32^P]dCTP. The PCR products were then subjected to single-strand conformational polymorphism (SSCP) analysis under two conditions (with or without 5% glycerol). The primers used to PCR–SSCP analysis were as follows; 5′-GCTGTGCATTGCAGCATTAT-3′ and 5′-CGGGAGGAGATAAACTA-3′ for exon 1; 5′-AAGATTTTTTCCTCTCTCC-3′ and 5′-GAGATGTCCATAAATTAATA-3′ for exon 2; 5′-GGTTTTTCCCTATACACTT-3′ and 5′-TACAGCAGCTTAATAGTTTTC-3′ for exon 3; 5′-GAAGTTTTCAAACCTGTCTC-3′ and 5′-CACTTTCAACTTCTCTGCTT-3′ for forepart of exon 4; and 5′-AACAAAGGGTGGAACAGCA-3′ and 5′-AAGAGCCAACAGCCTCT-3′ for the hind part of exon 4.

### Statistical analyses

Differences of the data for cell proliferation, chemoinvasion assay and tumour volume of nude mice was statistically assessed by an ANOVA test (Scheffe's analysis).

## RESULTS

Eleven genes were identified to be specifically down-regulated in advanced prostate cancers by the cDNA-RDA method and all of these were identical to known genes listed as follows; LIM protein (GenBank Accession #AF061258), cytoplasmic dynein light chain 1 (U32944), small subunit of calpain (X04106), acylamino acid-releasing enzyme (D38441), human X-box binding protein 1 (hXBP-1)(M31627), PRO1073 (AF113016), Lsm1 (AJ238094), 5-aminoimidazole-4-carboxamide ribonucleotide formyltransferase/inosine monophosphate cyclohydrolase (U37436), KIAA0088 gene (D42041), prostasin (L41351) and thyroid hormone-binding protein (J02783). On the other hand, all of seven genes up-regulated in advanced cancers were unknown.

**Figure 1:**
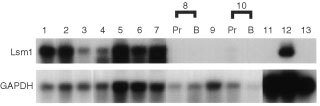
Northern blot analysis of human prostate total RNAs. Lanes 1 to 3, normal prostate tissues; lanes 4 to 7, organ-confined cancers from prostatectomized cases; lanes 8 to 10, refractory cancers from autopsy cases, Pr: primary site, B: metastatic focus to the bone; lane 11, DU145; lane 12, LNCaP; lane 13, PC3. Each lane was loaded with 10 μg of total RNA.

**Figure 2:**
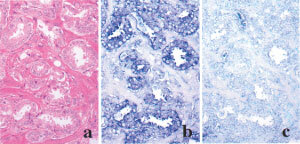
Lsm1 mRNA expression in serial sections of a prostate adenocarcinoma. (**a**) H&E. *In situ* hybridization with antisense (**b**) and sense (**c**) probes.

Among these, Lsm1 mRNA expression was detected by Northern blot analysis in three of three normal prostates, four of four organ-confined prostate cancers and in LNCaP cells, while no signals were found for three cases of advanced prostate cancers, including primary sites and bone metastatic foci, as well as DU145 and PC3 cells (Figure 1

). Lsm1 mRNA expression was detected in normal epithelial and adenocarcinoma cells in the human prostates by *in situ* hybridisation (Figure 2

). Lsm1 mRNA expression levels in LNCaP cells did not alter with growth in medium lacking androgens (data not shown).

**Figure 3:**
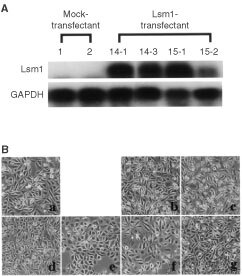
Establishment of PC3/Lsm1-transfectants. (**a**) Total RNAs were extracted from PC3, mock and Lsm1-transfectants and analysed for Lsm1 levels by Northern blotting. (**b**) Morphology of PC3 and transfectant cells. PC3 (a), mock1 (b), mock2 (c), Lsm1/14-1 (d), Lsm1/14-3 (e), Lsm1/15-1(f) and Lsm1/15-2 (g).

Four stable PC3/Lsm1-transfectants (#14-1, #14-3, #15-1 and #15-2) were established, which demonstrated no significant differences in morphology or cell proliferation in comparison with parent PC3 or PC3/mock-transfectants (Figures 3

and 4

**Figure 4:**
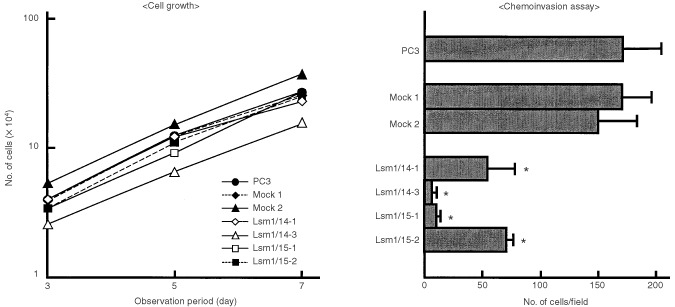
Data for cell growth and chemoinvasion assays of Lsm1-transfectants (means±s.d.). *Significantly different from the values for PC3, mock1 or mock2 at *P*<0.001.

). In contrast, significant reduction of invasive potential of all Lsm1-transfectants was observed in the Matrigel chemoinvasion assay (Figure 4). Dihydrotestosterone did not affect the growth of Lsm1-transfectants (data not shown).

Three out of four transfectants were able to form tumour masses in implantation sites of nude mice. The average time for tumour formation with the #15-1 transfectant was about 17 weeks while with the other transfectants (#14-1, #15-2) it was about 5 weeks. There were no obvious differences in the tumour volumes of each cells. Lymph node metastasis was reduced in #15-1 and #15-2 (Table 1

**Table 1:**
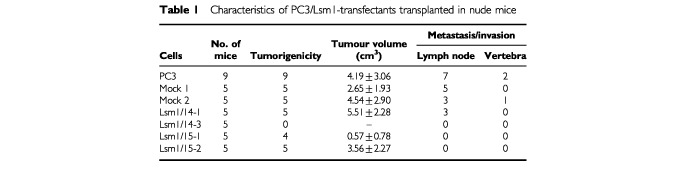
Characteristics of PC3/Lsm1-transfectants transplanted in nude mice

). The transfectant #14-1 showed lymph node metastases (three out of five mice) and marked reduction of Lsm1 gene expression in these metastatic foci and the primary tumours (implantation site) on *in situ* hybridization. The finding suggested that Lsm1 expression ceased in the #14-1 transfectant during the course of proliferation at the implantation site.

**Figure 5:**
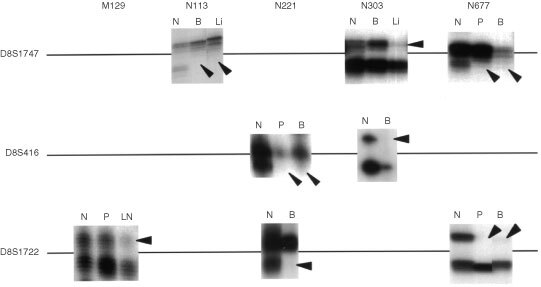
Autoradiograms illustrating the allelic losses on chromosome 8p11.2 in prostate cancers. Arrowheads indicate allelic loss. The primer sequences used were 5′-CCACCTCTGATATGCCAATCAAG-3′/5′-TGGTTTCCTAAAACTTCACCCG-3′ for D8S1747, 5′-GAGAATCGCTGGAACAGAGAAGG-3′/5′-GGCAGAGCTACCAAGAAACCAAAC-3′ for D8S416 and 5′-CCTTGCTGGGAATTGTG-3′/5′-AGCTGCCTGGCTAAGAG-3′ for D8S1722. N, normal tissues; P, primary site; LN, lymph node metastasis; B, bone metastasis; Li, liver metastasis.

D8S1747 and D8S416 markers were demonstrable located 700 and 350 kbp, respectively, downstream of the Lsm1 gene, while D8S1722 was present 120 kbp upstream of the gene. Almost all of the informative prostatectomised cases without neoadjuvant therapy showed allelic retention of D8S1747, D8S416 and D8S1722 loci, except for one lymph node metastatic focus (case #M129), whereas refractory cancers frequently showed allelic loss of these marker loci (Figures 5

and 6

**Figure 6:**
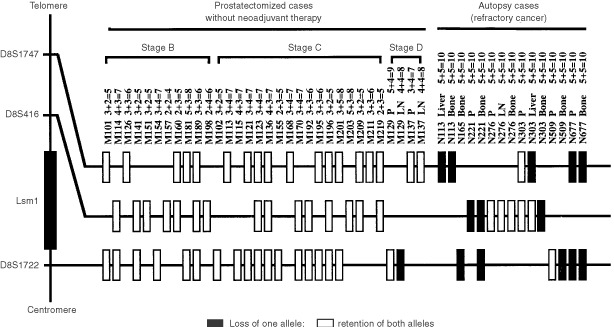
Summary of LOH for polymorphic markers surrounding the Lsm1 gene on chromosome 8p11.2 in a series of prostate cancers. P, primary site; LN, lymph node metastasis; Liver, liver metastasis; Bone, Bone metastasis. No mark indicates not informative. Numbers following the individual sample number represent their Gleason's score.

).

The available nucleotide sequence information of the GenBank accession number AP000065 and the data from LA (long and accurate) – PCR analysis for the intron 1 sequence revealed that Lsm1 is formed of four coding exons and three intervening introns spanning an area of 13 100 bp of genomic sequence on chromosome 8p11.2. The intron/exon splice sites and their flanking sequences are in agreement with the consensus splicing signals (Iida, 1990). No critical gene mutations were found in the open reading frame region of Lsm1 in 46 prostate cancer cases examined, including localized and refractory cases, by PCR–SSCP analysis.

## DISCUSSION

Lsm1 was first isolated from human pancreatic cancers as an up-regulated gene and termed cancer-associated Sm-like protein (Casm) (Schweinfest *et al*, 1997). Subsequently, Salgado-Garrido *et al* (1999) identified that Casm gene is identical to Lsm1 (like Sm protein 1). Eight Lsm proteins, Lsm1–Lsm8, have so far been reported in humans (Achsel *et al*, 1999; Salgado-Garrido *et al*, 1999). Seven of these, Lsm2–Lsm8, have been shown to interact with U6 snRNA and facilitate formation of U4/U6 RNA duplexes (Achsel *et al*, 1999). Recent reports demonstrated that yeast Lsm1 protein affects mRNA metabolism, particularly mRNA decapping and degradation (Boeck *et al*, 1998; Tharun *et al*, 2000) and it is possible that human Lsm1 regulates some specific genes related to tumour invasion or metastasis in prostate cancer cells. Further investigations are needed to clarify the molecular mechanisms of suppression of metastasis related to overexpression of the Lsm1 gene.

Although the number of samples was small and the quality of RNAs from refractory cancers due to sampling at autopsy was problematic, it is clear from the present transfection study that down-regulated expression of Lsm1 gene located at 8p11.2 is intimately involved in invasive and metastatic ability of human prostate cancers. The possible presence of putative tumour suppressor or metastasis suppressor genes in chromosome 8p11.2 in prostate cancers has been pointed out by several authors (Macoska *et al*, 1995; Crundwell *et al*, 1996; Prasad *et al*, 1998). Deletion of chromosome 8p11.2 has been frequently found in malignancies including stomach (Baffa *et al*, 2000), kidney (Schullerus *et al*, 1999), urinary bladder (Choi *et al*, 2000) and colorectal cancers (Chughtai *et al*, 1999), suggesting that Lsm1 gene is a candidate tumour suppressor or metastasis suppressor gene in common for a variety of human neoplasms.

The findings of a close association between down-regulation of Lsm1 and prostate cancer progression and the fact of abundant expression of Lsm1 in LNCaP but not in PC3 cells appear in conflict with the data reported by Schweinfest *et al* (1997). They also demonstrated diverse expression levels in many cancer cell lines. Regarding the two discrepancies, a possible cause of the former might be tissue-type specific Lsm1 expression. We have no explanation for the latter.

KAI1, a metastasis suppressor gene and a member of the membrane glycoprotein family, has been mapped to chromosome 11p11.2 and is known to contribute to metastasis of prostate cancer through down-regulation without gene mutations (Dong *et al*, 1995, 1996). Data on allelic loss of KAI1 region in prostate cancers are, however, conflicting, and it has been demonstrated that LOH is infrequent in American patients (Dong *et al*, 1996), while frequent in Japanese (Kawana *et al*, 1997). The standard form of CD44 is also a candidate metastasis suppressor gene located on chromosome 11p13, with potential involvement in prostate cancer progression through decreased expression (Gao *et al*, 1997). The present results demonstrated that Lsm1 suppressed prostate cancer metastasis through down-regulation, similar to the cases with the known metastasis suppressors, KAI1 or CD44, and we can speculate that the mechanism of decreased expression of this gene is reduction of gene dosage by allelic loss.

cDNA–RDA is a powerful and highly efficient method for identification of differentially expression of genes between two mRNA populations. However, it has limitations for enrichment of all differentially expressed genes during the procedure because of preferential amplification of some messages and the lack of Sau3A1 restriction sites in cDNAs. This might explain why genes known to be differentially expressed in advanced prostate cancer, such as KAI1 or CD44, were not isolated in the present study.

As shown in Figure 1, Lsm1 was here found to be expressed in LNCaP cells but not in PC3 and DU145 cells. These results suggest that Lsm1 expression may contribute to acquisition of the androgen-independent phenotype during prostate cancer progression. The lack of androgen receptors is the major cause of androgen-independency in PC3 and DU145 cells, with two factors thought to contribute the loss of androgen receptor expression; methylation of androgen receptor promoter and relative deficiency of transcription factors (Chlenski *et al*, 2001). As described above, Lsm1 might be involved in regulating some transcription factors related to androgen receptor expression but it may not interfere with the control of methylation levels in the promoter region. Hence it may be that overexpression of Lsm1 alone was not able to render PC3 cell androgen-dependent. The finding of high expression in LNCaP cells, which are derived from a lymph node metastasis, appears to contradict the conclusion of Lsm1 being a metastasis suppressor gene. However, an explanation for this may lie with alteration of the genes which are regulated by Lsm1. In fact, several genes have been identified to fluctuate with overexpression of Lsm1 using cDNA expression arrays (data not shown). Therefore, clarification of the signalling cascade regulated by Lsm1 may contribute to elucidation of the molecular mechanisms responsible for acquisition of metastatic ability in human prostate cancers.
